# Rhein sensitizes human pancreatic cancer cells to EGFR inhibitors by inhibiting STAT3 pathway

**DOI:** 10.1186/s13046-018-1015-9

**Published:** 2019-01-23

**Authors:** Lehe Yang, Shichong Lin, Yanting Kang, Youqun Xiang, Lingyuan Xu, Jifa Li, Xuanxuan Dai, Guang Liang, Xiaoying Huang, Chengguang Zhao

**Affiliations:** 1 Chemical Biology Research Center, School of Pharmaceutical Sciences, Wenzhou Medical University, Building 11, Chashan Street, University Town, Wenzhou, Zhejiang, 325035 People’s Republic of China; 2 Division of Pulmonary Medicine, The First Affiliated Hospital of Wenzhou Medical University, Key Laboratory of Heart and Lung, Wenzhou, Zhejiang, 325000 People’s Republic of China; 3 Department of Respiratory Medicine, Affiliated Yueqing Hospital, Wenzhou Medical University, Wenzhou, 325600 Zhejiang, People’s Republic of China; 4 Department of Ultrasonography, Yichun People’s Hospital, Yichun, Jiangxi 336000 People’s Republic of China

**Keywords:** Rhein, Pancreatic cancer, STAT3, EGFR, Inhibitor

## Abstract

**Background:**

Rhein is a lipophilic anthraquinone extensively found in medicinal herbs. Emerging evidence suggests that rhein has significant antitumor effects, supporting its potential use as an antitumor agent. The IL6/STAT3 signaling pathway has been suggested as an attractive target for the discovery of novel cancer therapeutics.

**Methods:**

The human pancreatic cancer cell lines AsPC-1, Patu8988T, BxPC-3 and PANC-1, and immunodeficient mice were chosen as models to study the effects of rhein. The potent antiproliferative and proapoptotic effects of rhein were examined by cell viability, cellular morphology, apoptosis and colony formation assays. The STAT3 luciferase report assay, immunostaining analysis and Western blot analysis revealed the inhibition of the IL6/STAT3 signaling axis.

**Results:**

Apoptosis was induced by adjunctive use of rhein with epidermal growth factor receptor (EGFR) inhibitors in pancreatic cancer cells as verified by cell apoptosis analysis and changes in the expression level of apoptotic/anti-apoptotic proteins BCL-2, BAX, Caspase 3 and Cl-PARP. Suppression of the phosphorylation of STAT3 and EGFR were also observed as a result of the treatment with a combination of rhein and EGFR inhibitors. Most interestingly, it was found that rhein considerably sensitized cells to erlotinib, thus suppressing tumor growth in PANC-1 and BxPC-3 xenograft models. The in vivo anti-tumor effect was associated with increased apoptosis and combined inhibition of the STAT3 and EGFR pathways in tumor remnants.

**Conclusions:**

Rhein sensitizes human pancreatic cancer cells to EGFR inhibitors through inhibition of STAT3. Taken together, the results indicate that rhein offers a novel blueprint for pancreatic cancer therapy, particularly when combined with EGFR inhibitors.

**Electronic supplementary material:**

The online version of this article (10.1186/s13046-018-1015-9) contains supplementary material, which is available to authorized users.

## Background

Pancreatic cancer (PC) is one of the most lethal malignancies, with a median survival of less than 6 months after diagnosis, and a 5-year survival rate below 6% [1]. Surgery is considered the most effective treatment for PC, unfortunately, patients are diagnosed at an advanced stage, and only 20% of patients are suitable for surgical resection based on disease staging [2, 3]. Currently, chemotherapy is still an important treatment of metastatic or advanced-stage PC, which prevents recurrence and prolongs patient survival [4–6]. Epidermal growth factor receptor (EGFR) is a member of the tyrosine kinase receptors (TKR) family, and it regulates cell proliferation, apoptosis, differentiation, migration and angiogenesis [7]. The activated EGFR stimulates various downstream signaling pathways, including the Ras/Mitogen-activated protein kinase kinase kinase (Raf, MAP3K)/Mitogen-activated protein kinase kinase (MEK, MAP2K)/ Mitogen activated protein kinase (MAPK), Phosphoinositide 3-kinase (PI3K)-Protein kinase B (AKT) and the Janus Kinase (JAK)/signal transducer and activator of transcription (STAT) signaling pathways, leading to tumor development and metastasis [8, 9]. As a result, EGFR has become an attractive molecular target for cancer therapy, including PC. EGFR inhibitors according to their site of action can be divided into two categories: one is EGFR small molecule tyrosine kinase inhibitors (TKIs), including the currently used erlotinib, gefitinib, afatinib and osimertinib (AZD9291) [10, 11]. The other is the extracellular EGFR monoclonal antibodies that specifically bind EGFR (EGFR-mAb), such as etuximab and panitumumab, etc. [9, 12]. The EGFR inhibitor erlotinib (Tarceva, OSI Pharmaceuticals, Inc., Melville, NY, USA) is the US Food and Drug Administration (FDA) approved it in combination with gemcitabine for treatment of locally advanced, unresectable, or metastatic PC [13].

Although targeted drugs against EGFR have been increasingly developed for the treatment of PC, unfortunately, treated patients develop resistance, limiting the benefit to patients and posing a challenge to oncologists. Despite excellent initial clinical responses, nearly all responding patients invariably develop secondary resistance after a median period of about 10–16 months [14, 15]. As an important member of the STAT family of transcription factors, STAT3 is linked to malignant transformation and tumor progression [16, 17]. Recent studies underscore the involvement of STAT3 in PC through its regulation of tumor cell proliferation, survival, tumor invasion and angiogenesis [18]. STAT3 has been shown to have roles in mediating PC resistance [19]. Our previous research studies have shown that STAT3 activation is an important alternative pathway mediating the EGFR/MEK inhibitor resistance [19, 20]. Other research groups also found that in cancer cells the EGFR inhibitors erlotinib and dacomitinib can activate the interleukin-6 (IL6) / JAK / STAT3 signaling pathway, thereby leading to drug resistance [21, 22]. In the treatment of metastatic liver cancer with the EGFR inhibitor cetuximab, the tumor drug resistance is also associated with activation of STAT3 [23]. Therefore, the feedback activation of the STAT3 signaling pathway is an important mechanism of resistance to EGFR inhibitors.

**Fig. 1:**
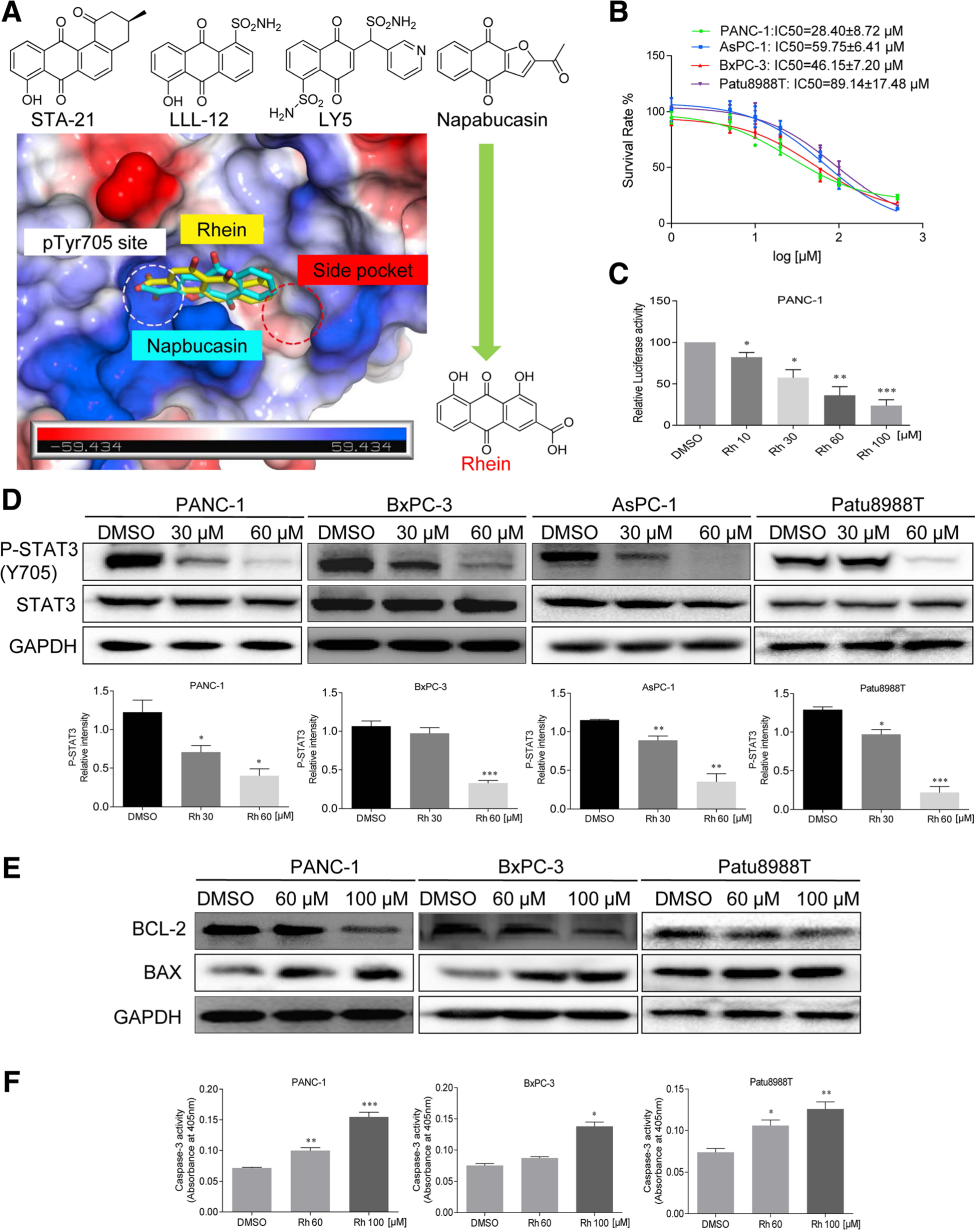
Rhein inhibits phosphorylation of STAT3 on Tyr705 and induces apoptosis in pancreatic cancer cells. **a** Docking of rhein and napabucasin to the STAT3 SH2. **b** The effects of rhein on cell viability in pancreatic cancer cells. Pancreatic cancer cells were treated with rhein at different concentration ranges as indicated for 48 h, then cell viability was evaluated by MTT assay, the IC_50_ is indicated. **c** PANC-1 cells were transfected with luciferase reporter gene plasmid and treated with rhein for 24 h. The results were normalized to the Renilla luciferase activity. **d** Rhein inhibited the phosphorylation of STAT3 Tyr705 in a dose-dependent manner. **e** Western blot analysis of the expression of apoptosis-associated proteins in cells treated with rhein. Cells were treated with rhein at different concentrations as indicated for 36 h, the cell lysates were processed for Western blot analysis of the protein expression of BCL-2 and BAX. GAPDH is used as a loading control. **f** Pancreatic cancer cells were treated with rhein for 36 h and assayed for caspase-3 activity. The statistical data are presented as mean ± SD from three independent experiments. *p < 0.05, **p < 0.01, ***p < 0.001

Recent studies have focused on the antitumor properties of natural products due to their confirmed pharmacological properties and few side effects [24]. Rhein (4, 5-dihydroxyanthraquinone-2-carboxylic acid, Fig. 1a) is an active ingredient present in various medicinal herbs, such as rhubarb (*Rheum rhabarbarum*) which exerts antitumor effects, by inhibiting tumor cell proliferation, invasion and metastasis, as well as promoting apoptosis. More recently, several studies have reported various mechanisms and pathways mediating the antitumor effects of rhein, but the direct molecular targets and specific mechanism remain unclear [25]. In this study, we elucidated the specific molecular mechanism whereby rhein exerts antitumor effects by inhibiting STAT3. Furthermore, based on the relationship between the EGFR and STAT3 signaling pathways, we have designed a rational combination of drugs for the treatment of patients with PC.

## Methods

### Antibodies and reagents

The antibodies against P-STAT3, STAT3, P-EGFR, EGFR, Lamin B, Cleaved poly ADP-ribose polymerase (Cl-PARP), B-cell lymphoma 2 (BCL-2), BCL-2-associated X protein (BAX), Glyceraldehyde 3-phosphate dehydrogenase (GAPDH), horseradish peroxidase (HRP)-conjugated donkey anti-rabbit IgG and HRP-conjugated goat anti-mouse IgG were purchased from Santa Cruz Biotechnology Inc. (Dallas, TX, USA). Methylthiazolyldiphenyl-tetrazolium bromide (MTT) and dimethyl sulfoxide (DMSO) were purchased from Sigma-Aldrich Co. (St Louis, MO, USA). The caspase-3 colorimetric assay kit was purchased from Abcam Co. (Cambridge, MA, USA). The Annexin V-FITC apoptosis Detection Kit I and propidium iodide (PI) were purchased from BD Pharmingen (Franklin Lakes, NJ, USA). The Dual-Luciferase Report Assay Kit was obtained from Promega Biotech Co., Ltd. (Madison, WI, USA). Rhein, erlotinib, afatinib and gefitinib were purchased from Aladdin biochemical technology company (Shanghai, China). The compounds used in vitro were dissolved in DMSO. A Bradford protein-assay kit, polyvinylidene fluoride membrane, and enhanced chemiluminescence kit were obtained from Bio-Rad Laboratories (Hercules, CA, USA). A protease phosphatase-inhibitor mixture was obtained from Applygen Technologies (Beijing, China). Acrylamide (30%), Coomassie Brilliant Blue, tetramethylethylenediamine, Tris-glycine, sodium dodecyl sulfate, prestained protein marker, and nonfat dry milk were from Bio-Rad Laboratories.

### Cell culture

Human pancreatic cancer cell lines BxPC-3, PANC-1, Patu8988T and AsPC-1 were obtained from Shanghai Institute of Biosciences and Cell Resources Center (Chinese Academy of Sciences, Shanghai, China). AsPC-1 and BxPC-3 cells were grown in McCoy’s 5A medium (Gibco) supplemented with 10% fetal bovine serum (FBS, Gibco), PANC-1 and Patu8988T cells were cultured in Roswell Park Memorial Institute (RPMI)-1640 media (Gibco) with 10% FBS. All the cells were cultured in a humidified cell incubator with an atmosphere of 5% CO_2_ at 37 °C.

### Docking of rhein and napabucasin to the STAT3 SH2

The STAT3 SH2 crystal structure was obtained from the PDB (PDB code: 1BG1), and used as the protein target for docking simulation [26]. AutoDockTools (ADT) 1.5.6 was employed for the preparation of input PDBQT files of ligands and protein receptor STAT3 SH2 as described in the instruction [27]. Docking simulations was performed using AutoDock Vina 1.0.2 [28]. The docking grid was a 3-D 24 Å × 16 Å × 24 Å box, which encompassed the entire binding region. The Vina docking parameters were as follows: a receptor binding center of x = 103, y = 72.5, and z = 64.1; an energy range of 5 Kcal/mol; an exhaustiveness of 25; and an output number of 5 binding modes ranked by highest binding affinity. The binding modes were viewed and analyzed using the ADT tool, and the top binding modes with best binding affinity (most negative binding energy) were selected.

### Western blot analysis

Cancer cell lines were seeded in 6-well plates at a density of 500,000 cells per well and then incubated overnight. Different dilutions of the corresponding drugs were added into the 6-well plates. Treated cells were washed with phosphate-buffered saline (PBS) and harvested using ice-cold RIPA lysis buffer with 1% PMSF. Proteins were separated by 10% sodium dodecyl sulfate-polyacrylamidegel (SDS-PAGE) and then transferred onto a polyvinylidene fluoride (PVDF) membrane blocking with 5% skim milk. The blots were incubated with specific primary antibodies. After washing, the membranes were incubated with the relevant secondary antibodies, visualization of bands was by enhanced chemiluminescence.

### MTT cytotoxicity assay

MTT cytotoxicity assay was utilized to measure human pancreatic cancer cells cytotoxicity and viability. Cells (3 × 10^3^ cells/well) were plated in 96-well plates and allowed to attach overnight. After appropriate treatment, the cells were treated with MTT solution (5 mg/mL) for 4 h at 37 °C. The formazan crystals were dissolved in 150 μL DMSO and the optical density (OD) was measured using a Microplate Reader at 490 nm. The cell viability was calculated according to the following formula: viability = (average OD values of treatment wells/average OD values of vehicle control wells) × 100%. And, half-maximal inhibitory concentration (IC_50_) values were determined by GraphPad Pro Prism 7.0. CompySyn is used for determining synergism and antagonism, where combination index (CI) < 1, =1, and > 1 indicates synergism, additive effect and antagonism, respectively (ComboSyn Inc. and PD Science LLC) [29].

### Caspase-3 activity assay

Caspase-3 activity was assessed using a caspase-3 colorimetric assay kit (Abcam, Cambridge, MA, USA) according to the manufacturer’s protocol. Briefly, the pancreatic cancer cells were collected and resuspended in lysis buffer. Following incubation for 10 min on ice, cell lysate was centrifuged at 12000 g for 10 min at 4 °C, and the protein concentration in the supernatants was measured using the Bradford dye method. The supernatants were incubated with reaction buffer containing 200 μM devd-p-nitroanilide, for caspase-3 in a caspase assay buffer at 37 °C with 10 mM dithiothreitol for 2 h. Caspase-3 activity was determined by measuring the absorbance at 405 nm. Each experiment was done in triplicate for three independent experiments.

### Colony formation assay

The PANC-1 and AsPC-1 cells were seeded at 1000 cells/well on 6-well plates at 37 °C in 5% CO_2_ atmosphere overnight. DMSO (Control), 60 μM rhein, EGFR inhibitors (5 μM erlotinib or 2.5 μM afatinib) or the combination was added to the cells for 7 days. The culture medium was replaced by the fresh medium every two days to keep cells growing for 7 days. Colonies were washed with PBS, fixed with 4% paraformaldehyde at room temperature for 15 min, washed with purified water for 3 times and stained with Crystal violet for 10 min. Each experiment was done in triplicate for three independent experiments.

### Hoechst 33258 staining

A Hoechst 33258 assay kit (Beyotime Institute of Biotechnology, China) was used to detect apoptosis in pancreatic cancer cells. Cells were seeded in 6-well plates and incubated with DMSO (Control), 60 μM rhein, 5 μM erlotinib or the combination. After 36 h, cells were washed in PBS and fixed in freshly prepared 4% paraformaldehyde for 15 min. Cells were then washed with PBS again, incubated with Hoechst 33258 staining solution for 20 min, and washed with PBS before antifade mounting medium was added. Apoptotic cells were detected using a fluorescence microscope.

### Cell apoptosis assay

Apoptosis was determined using an apoptosis detection Kit (BD Biosciences, USA). Briefly, human pancreatic cancer PANC-1 cells were seeded into 6-well plates and allowed grown to 80% confluency in complete medium, then cells were treated with DMSO (Control), 60 μM rhein, 5 μM erlotinib or the combination for 36 h to evaluate the effects of those compounds on apoptosis. Cells were collected, washed twice in ice-cold PBS, and then resuspended in binding buffer according to the instructions of the apoptosis Kit. The treated cells (as described above) were simultaneously incubated with fluorescein-labeled Annexin V and PI. Annexin V-binding buffer was then added to the mixture before fluorescence was measured on a FACSCalibur (BD Biosciences; Baltimore, MD, USA). Data were analyzed using Flowjo software.

### STAT3 luciferase report assay

The STAT3 luciferase reporter plasmid (pGLSTAT3-Luc) was used to detect STAT3 activation. Briefly, human pancreatic cancer PANC-1 cells were seeded in 24-well plates 24 h before transfection. Then, the cells were co-transfected with pGLSTAT3-Luc and pRL-TK (a plasmid encoding Renilla luciferase) using Lipofectamine 3000 (Invitrogen, Carlsbad, CA, USA) for 6 h. Finally, the cells were treated with the indicated concentrations of rhein for 24 h. Luciferase activity was assessed by SpectraMax ID3 (Molecular Devices, San Jose, CA, USA). The inhibition of STAT3 activation by rhein was calculated as the ratio between the value of firefly and Renilla luciferase activity. Each experiment was done in triplicate for three independent experiments.

### Immunostaining of P-STAT3

Cells were cultured in 6-well plates. After treatment, the cells were fixed by 4% paraformaldehyde for 20 min at 4 °C. The cells were then washed three times with PBS and incubated in 1% Triton X-100 for 15 min and 1% BSA for 1 h at room temperature. Cells were then incubated overnight at 4 °C on addition of anti-P-STAT3 primary antibody solution (1:300 in 3% BSA). After rewashed in PBS, the cells were allowed to react with PE-labeled secondary antibody (1:300 in 3% BSA) for 1 h in a dark room and counterstained 4,6-diamidino-2-phenylindole dihydro-chloride (DAPI) for 5 min. Images were captured under the fluorescence microscope.

### Xenograft models

The mouse studies were conducted in compliance with the Wenzhou Medical University’s Policy on the Care and Use of Laboratory Animals. Protocols for the mouse studies were approved by the Wenzhou Medical College Animal Policy and Welfare Committee. Five-week-old athymic BALB/c female mice (18–20 g) were used for the in vivo experiments. The mice were housed at a constant room temperature with a 12/12-h light/dark cycle and fed a standard rodent diet and given water ad lib. Human pancreatic cancer cells were injected subcutaneously into each mouse,including PANC-1 (1 × 10^7^) and BxPC-3 (5 × 10^6^), respectively. Upon attaining an appropriate tumor volume (approximately 7–10 days post-implantation), the mice were randomized into 4 groups, and intraperitoneal injection with 60 mg/kg rhein, 10 mg/kg erlotinib or the combination. Control mice received liposome vehicle in PBS. The tumor volumes were determined by measuring length (L) and width (W) and calculating volume(V = 0.5 × L × W^2^) at the indicated time points. At the end of study, the mice were sacrificed, and tumors harvested and weighed. Each sample was cut in halves; one half was preserved in 4% paraformaldehyde and the other half was flash-frozen in liquid nitrogen and stored at − 80 °C until further use (histological and protein expression analyses).

### Hematoxylin and eosin (H&E) staining

The heart, lung, kidney, and liver tissues of four groups were fixed in 4% paraformaldehyde and embedded in paraffin. The paraffin tumor tissue sections (5 μm) were deparaffinized and rehydrated and then stained with eosin and hematoxylin. The images were captured using a light microscope.

### Statistical analysis

Data were expressed as mean ± standard deviation (SD) of three independent experiments. The statistical differences between different groups were analyzed by the student’s t-test or one-way analysis of variance in GraphPad Pro 7.0 (GraphPad, San Diego, CA). *P* values less than 0.05 (*p* < 0.05) were considered indicative of significance.

## Results

### Rhein suppresses constitutive STAT3 tyrosine phosphorylation and induces apoptosis in pancreatic cancer cells

Based on the important role of STAT3 in the process of acquisition of resistance to EGFR inhibitors and the structural similarity of rhein with the four known inhibitors of STAT3, namely napabucasin, STA-21, LLL12 and LY5 [19], computer-aided molecular docking results showed that rhein may combine directly with the STAT3 protein and affect the phosphorylation of the Y705 active site (Fig. 1a). We then examined the effect of rhein on the growth of PC cell lines. Treatment of PANC-1, BxPC-3, AsPC-1 and Patu8988T cells with various concentration of rhein for 48 h, we observed a dose-dependent inhibition of cell growth in all the cell lines (Fig. 1b). Additionally, PC cells were exposed to the indicated concentrations of rhein to confirm the inhibition of STAT3 activation. As predicted, the results of STAT3 luciferase reporter and Western blot analysis demonstrated that rhein specifically blocked STAT3 signaling (Fig. 1c and Additional file 1: Figure S1A). Notably, rhein inhibited STAT3 phosphorylation at tyrosine 705 in a dose-dependent manner. Hovever, the total STAT3 protein level was not affected by rhein (Fig. 1d). Previous findings, including our own, have established that inhibition of constitutively activated STAT3 causes apoptosis in cancer cells [20]. Here, we also found that decrease in STAT3 phosphorylation was concomitant with the changes in expressions level of BCL-2, BAX and Caspase-3. (Fig. 1e-f and Additional file 1: Figure S1B).

### Rhein treatment reduced nuclear localization of STAT3

**Fig. 2:**
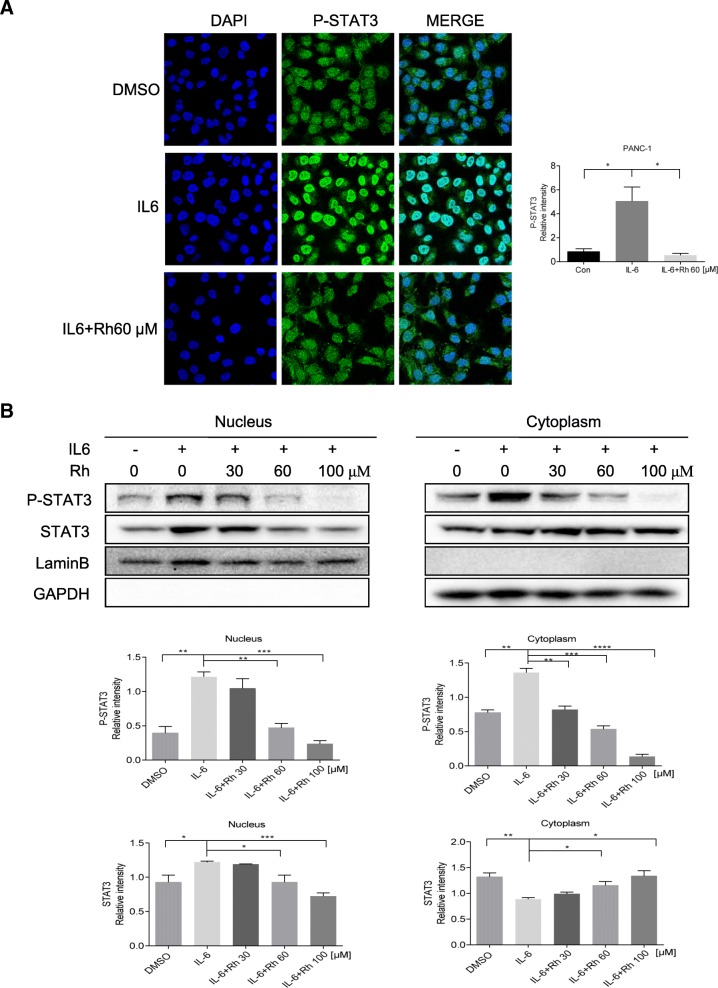
Rhein inhibits interleukin 6 (IL6) induced activation of STAT3 in pancreatic cancer cells. **a** PANC-1 cells were treated with rhein for 24 h. For stimulation, cells were exposed to IL6 (20 ng/ml) for 15 min at the end of incubation period. The staining for phosphorylated STAT3 (P-STAT3) and nuclei is shown as green and blue fluorescence. Immunofluorescence was performed as described in materials and methods to analyze the expression levels of P-STAT3. Confocal microscopy representative of numerous randomly selected microscopic fields from at least 3 independent experiments are shown. **b** PANC-1 cells were treated with rhein for 24 h, followed by stimulation with IL6 (20 ng/ml) for 30 min, and the cytoplasmic and nuclear extracts were subjected to immunoblotting to detect the distribution of P-STAT3 and STAT3. Experiments were performed in triplicate and were independently repeated three times. *p < 0.05, **p < 0.01, ***p < 0.001,****p<0.0001

We next determined whether rhein treatment affected nuclear translocation of STAT3 in PANC-1 cells. As anticipated, exposure of PANC-1 cells to 20 ng/mL IL6 for 15 min resulted in increased nuclear level of phosphorylated STAT3 (P-STAT3) as evidenced by more intense green staining compared with unstimulated PANC-1 cells. IL6 treatment resulted in the translocation of P-STAT3 (green fluorescence) from the cytoplasm into the nucleus (blue fluorescence, DAPI stained). As expected, rhein can inhibit this translocation (Fig. 2a). Moreover, as shown in Fig. 2b, STAT3 nuclear translocation was largely inhibited when exposed to rhein. These results further demonstrated inhibition of IL6-induced nuclear translocation of P-STAT3 to the mucleus by rhein in pancreatic cell lines.

### Synergistic effects of rhein and erlotinib/afatinib in human pancreatic cell lines

**Fig. 3:**
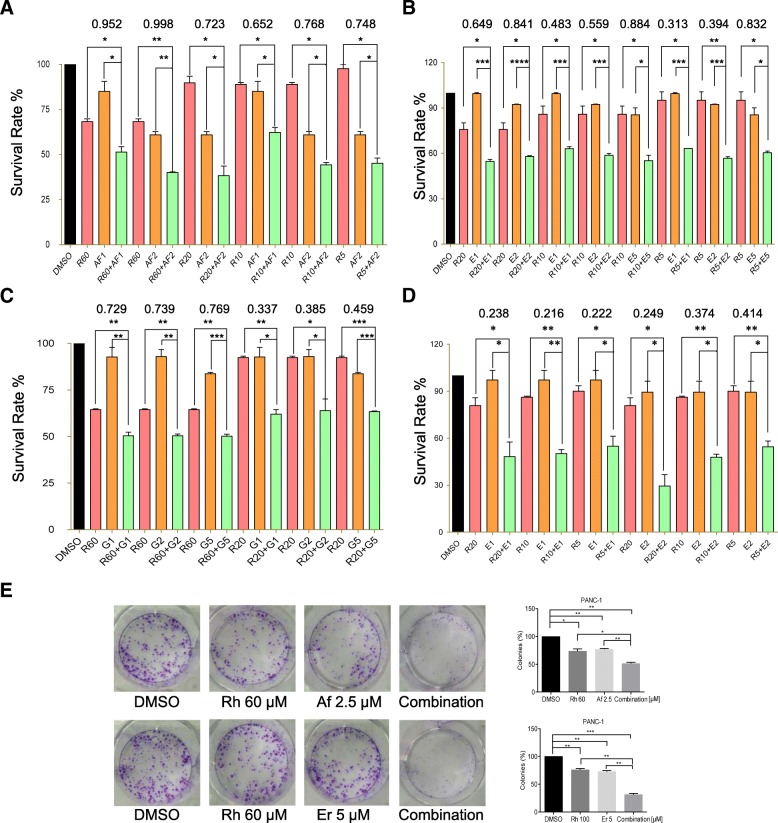
Combined rhein and EGFR inhibitors synergistically suppress pancreatic cancer cell proliferation. **a** PANC-1 cells were treated with serial dilutions of rhein, the EGFR inhibitor afatinib or the combination of rhein plus afatinib. Cell viability was measured after 2 days of treatment by the MTT assay. Survival rates were plotted using the GraphPad Prism software. CompySyn was used for determining the combination index. **b** The PANC-1 cells were treated with rhein, erlotinib or the combination. **c** The PANC-1 cells were treated with serial dilutions of rhein, gefitinib or the combination. **d** The AsPC-1 cells were treated with serial dilutions of rhein, erlotinib or the combination. **e** Colony forming assay in PANC-1 cells. Data are expressed as mean ± SD. Experiments were performed in triplicate and were independently repeated three times. *p < 0.05, **p < 0.01, ***p < 0.001, ****p<0.0001

STAT3 activation was recently suggested as a potential predictive marker for resistance to EGFR TKI therapies in patients with metastatic colorectal cancer (mCRC) and non-small cell lung cancer (NSCLC), given the correlation between the activation of STAT3 and resistance of cells to EGFR TKI therapy [30, 31]. To test whether combined treatment with rhein and erlotinib/afatinib provides an advantage compared to single treatment with each drug. Inhibition of cell growth was assessed by the MTT assay. We treated PC cells with erlotinib/afatinib either alone or in combination with rhein. The combination treatment decreased cell viability much more robustly than either agent alone, with combination index CI < 1 (Fig. 3a-d and Additional file 2: Figure S2). In addition, PC cells showed a decreased ability to recover and form colonies following the combination treatment with rhein and erlotinib/afatinib, these results indicated that the combined treatment of pancreatic cell lines with rhein and erlotinib/afatinib was more effective compared with the action of each individual drug (Fig. 3e and Additional file 1: Figure S1C).

### Combined treatment with Rhein and erlotinib/afatinib efficiently suppresses phosphorylation of both STAT3 and EGFR in human pancreatic cell lines

**Fig. 4:**
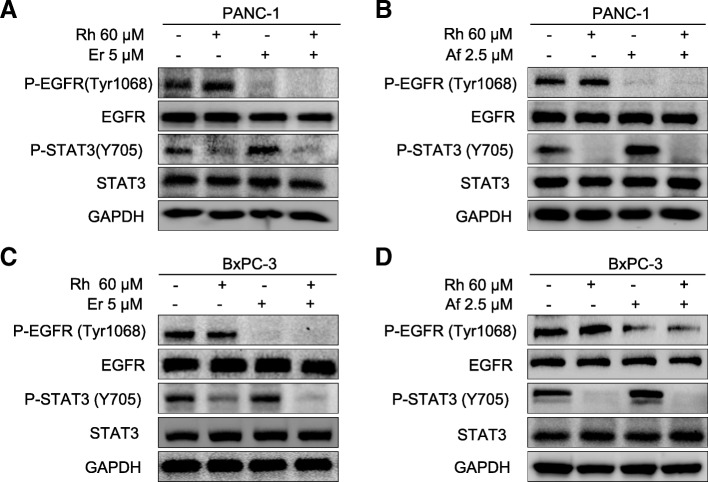
Inhibition of STAT3 by rhein blocks EGFR inhibitors activated STAT3. **a** PANC-1 cells were treated with DMSO (Control), 60 μM rhein, 5 μM erlotinib or the combination of both inhibitors (rhein + erlotinib). Whole-cell protein extracts were analyzed by Western blotting with the indicated antibodies. GAPDH antibody was used as loading control. Figures are representative of three independent experiments. **b** The PANC-1 cells were treated with DMSO (Control), 60 μM rhein, 2.5 μM afatinib or the combination. **c** The pancreatic cell lines BxPC-3 were treated with DMSO (Control), 60 μM rhein, 5 μM erlotinib or the combination of both inhibitors (rhein + erlotinib). **d** The BxPC-3 cells were treated with DMSO (Control), 60 μM rhein, 2.5 μM afatinib or the combination

Mechanistically, to further investigate the synergistic interaction between rhein and erlotinib/afatinib, we evaluated the molecular changes in PC cells after treatment with rhein and erlotinib/afatinib either individually or in combination. As shown in Fig. 4, treatment with erlotinib/afatinib alone resulted in an increased level of P-STAT3 (Y705), whereas treatment with rhein alone inhibited P-STAT3 in both cell lines, as expected. The combined treatment blocked the erlotinib/afatinib induced STAT3 activation and P-EGFR (Fig. 4).

### Synergistic effects of rhein and erlotinib on apoptosis

**Fig. 5:**
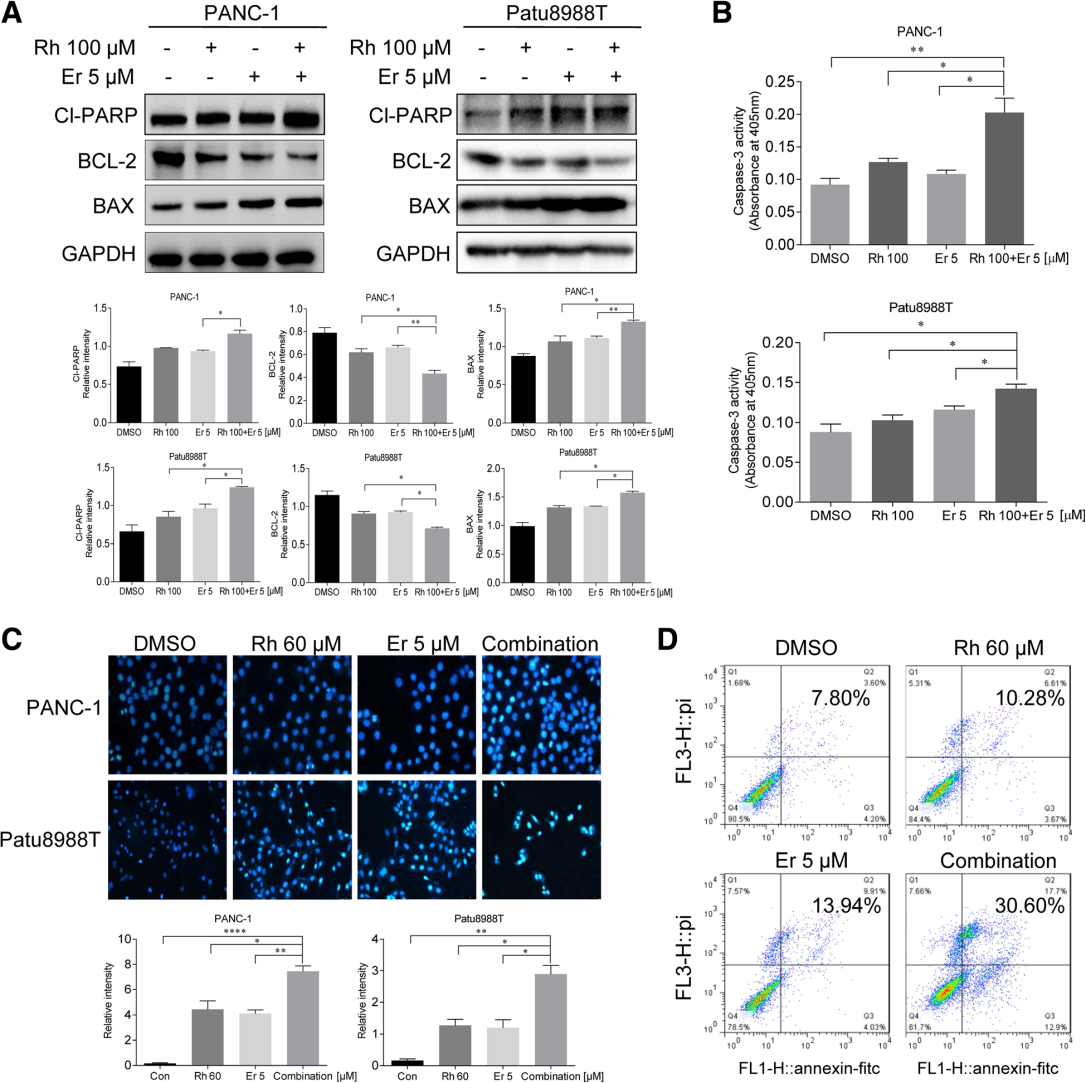
Combined rhein and EGFR inhibitors induce apoptosis in pancreatic cancer cell. **a** The indicated PANC-1 and Patu8988T cells were treated with DMSO (Control), 100 μM rhein, 5 μM erlotinib or the combination of both inhibitors. Whole-cell protein extracts were analyzed after 36 h of treatment by Western blotting with the indicated antibodies. GAPDH antibody was used as loading control. Results are representative of three independent experiments. **b** Pancreatic cancer cells PANC-1 and Patu8988T were treated with rhein and erlotinib 36 h and assayed for caspase-3 activity. **c** PANC-1 and Patu8988T cells were treated with DMSO (Control), 60 μM rhein, 5 μM erlotinib or the combination 36 h. The viable cells showed normal-shaped nuclei that were faintly stained with Hoechst 33258. The apoptotic cells exhibited shrunken nuclei with evidence of chromatin condensation. The results are representative of three replicate experiments (magnification, × 200). **d** Representative images for cell apoptosis stained with Annexin V-fluorescein isothiocyanate/propidium iodide (FITC/PI). PANC-1 cells were treated with rhein, erlotinib or the combination of both inhibitors for 24 h, then cells were stained with Annexin V-FITC/PI and analyzed by flow cytometry as described in the methods section. The statistical data are presented as mean ± SD from three independent experiments. *p < 0.05, **p < 0.01, ***p < 0.001, ****p<0.0001

To investigate the pharmacodynamic effect of combining rhein and erlotinib on cell apoptosis, PANC-1 and Patu8988T cells were exposed to monotherapy agents, or the combination therapy. Enhanced apoptosis in PC cells was detected in rhein and erlotinib treated groups as demonstrated by cell apoptosis analysis and changes in expression level of the apoptotic/anti-apoptotic proteins BCL2, BAX, Cl-PARP and Caspase 3. (Fig. 5a-b). Furthermore, the morphological features of apoptotic cells were visualized by Hoechst 33258 staining. Rhein and erlotinib were used to treat PC cells for 36 h, and then the cells were stained with Hoechst 33258. The percentage of apoptotic cells induced by rhein and erlotinib combined or alone were significant compared with the untreated control group. Furthermore, the apoptotic rate of the combined treatment was greater than those of each individual treatment (Fig. 5c). Additionally, we used an alternative assay for apoptosis based on changes in Annexin A5 (ANXA5) staining as monitored by flow cytometry. Here, cells were treated with Rhein alone, erlotinib alone or the combination. ANXA5 binding/flow cytometric analysis showed that higher percentages of PANC-1 cells undergo apoptosis in response to the concurrent inhibitory effects of rhein and erlotinib compared to that of either rhein or erlotinib alone (Fig. 5d).

### Anti-tumor activity of rhein and EGFR inhibitors erlotinib in vivo

**Fig. 6:**
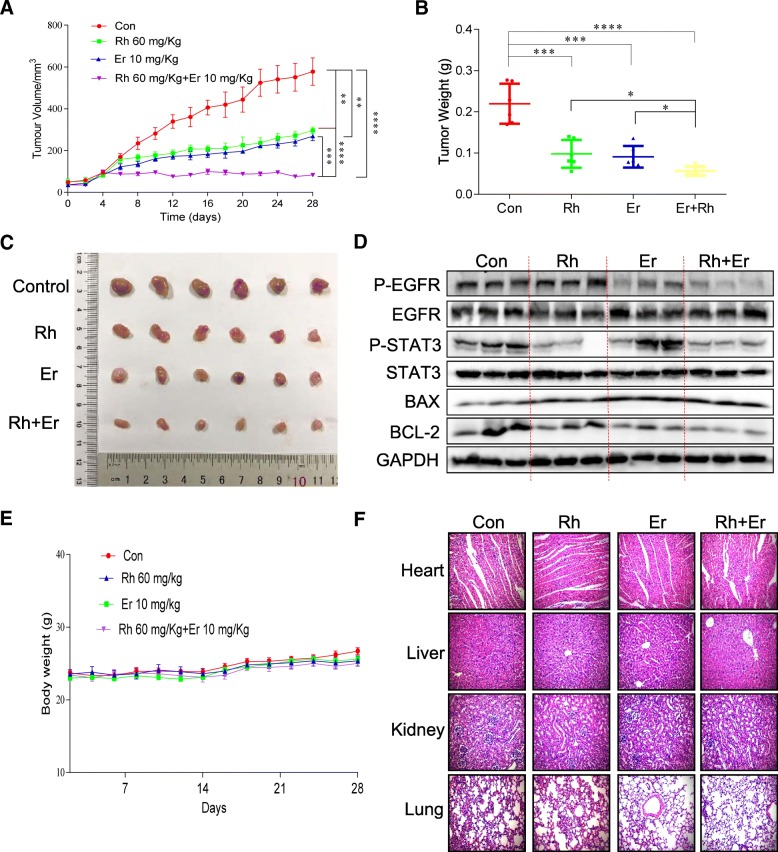
Combined treatment of rhein and EGFR inhibitors inhibit tumor growth in xenograft mouse model. **a** Antitumor efficacy of rhein and erlotinib in the PANC-1 xenograft mouse model. BALB/c mice (*n* = 6) were treated with DMSO (Control), 10 mg/kg erlotinib, 60 mg/kg rhein, or the combination. Tumor volumes were recorded every 2 days. **b** Comparison of the final tumor weights in each group after the 28-day treatment of erlotinib and rhein. Numbers in columns indicate the mean tumor weight in each group. **c** Photographs of tumors in each group. **d** Western blot analysis of tumor lysates for phosphorylated EGFR (P-EGFR), phosphorylated STAT3 (P-STAT3), BAX and BCL-2. GAPDH was used as loading control. **e** Body weight of mice. **f** No histological abnormalities were observed in kidney, liver, lung and heart in the rhein and erlotinib treated groups. Heart, kidney, lung and liver from the four groups were sectioned at 5 μm, and the slides were stained with hematoxylin and eosin (H&E) (*n* = 5 in each group). All images were captured using an optical microscope with 200× magnification. All images are representative of three independent experiments. The level of significance is indicated by *P < 0.05, **P < 0.01, ***P < 0.001, and ****P < 0.0001

To determine whether rhein and an EGFR inhibitor can synergistically inhibit the growth of cancer cells in vivo, rhein and the EGFR inhibitor erlotinib were selected for further evaluation of their in vivo antitumor efficacy in a human PC cells xenograft mouse model (Fig. 6 and Additional file 3: Figure S3). BALB/c nude mice (*n* = 6/group) were administered rhein at 60 mg/kg, erlotinib at 10 mg/kg or the combination (every two days). The tumor weight and volume were measured. The results indicated that rhein, erlotinib and the combination significantly inhibited tumor growth compared to the vehicle group (Fig. 6a-c). Remarkably, the expression of P-STAT3 and P-EGFR were downregulated after rhein and erlotinib treatment. In addition, the combination of rhein and erlotinib significantly decreased BCL-2 level and increased the BAX level compared to the other three groups (Fig. 6d). Moreover, no significant loss of body weight and major toxicities in any of the treatment groups occurred (Fig. 6e-f).

## Discussion

Although many patients are sensitive to EGFR inhibitors in the initial treatment, some patients will develop resistance to them due to EGFR secondary mutations. Afatinib and osimertinib have been expected to overcome the EGFR mutation mediated acquisition of resistance to first-generation reversible EGFR TKIs [10, 11, 32]. However, a recent phase III study of afatinib failed to show overall survival improvement in gefitinib or erlotinib resistant patients, with response rates below 10% [33]. There are other possible resistance mechanisms besides EGFR secondary mutations, such as activation of downstream signaling, cell transformation, epithelial-mesenchymal transition (EMT) and alternative activation pathways [34]. Although many studies have identified the mechanisms of resistance to EGFR inhibitors, the molecular mechanisms underlying the acquisition of TKI resistance are still not fully understood [14, 15]. Accordingly, a rational framework for its treatment needs to be established to standardize clinical studies, which in recent years, have increasingly suggested the importance of the activation of alternative pathways in the acquisition of resistance to EGFR TKI [35, 36].

Several studies have implicated STAT3 activation in the resistance to EGFR inhibitors [21, 22]. In addition, elevated levels of STAT3 have been reported in several drug resistant cancer cells where inactivation of STAT3 reversed the drug resistant phenotype [23]. Activated STAT3 (P-STAT3) status may contribute to the selection of patients eligible for anti-EGFR–based therapies. Emerging evidence indicates that rhein has antitumor activity and can attenuates drug resistance in human ovarian cancer cells [37]. Rhein also inhibits AlkB repair enzymes and sensitizes cells to methylated DNA damage [38]. The rhein-derived compound AQ-101 represents a potentially new, safe anti-tumor drug providing a novel strategy for targeting MDM2 [39]. In this study, we found that rhein significantly enhanced the effect of erlotinib and afatinib on PC by inhibiting STAT3 in vitro (Fig. 1, 2, 3, 4, 5) and in vivo (Fig. 6). Consistent with our results, molecular studies indicated that diacerein induced apoptosis was associated with inhibition of IL6/STAT3 signaling [40, 41].

To date, no direct STAT3 inhibitor has been approved for clinical use. Therefore, there is an urgent need for an effective and safer therapeutic STAT3 inhibitors to treat PC. Noteworthy, the antiproliferative efficacy of rhein was lower (higher IC50) than that of the known STAT3 inhibitors. Rhein is a lipophilic anthraquinone extensively found in medicinal herbs *Rheum rhabarbarum*., *Cassia tora* L. etc., which have been used medicinally for more than 1000 years [38]. In addition, diacerein, which is known to be completely metabolized into rhein by humans and animals, is clinically prescribed for the treatment of osteoarthritis [40, 41]. Moreover, we also found rhein has few side effects on the mouse body at the therapeutic concentration used in this study. Thus, the synergistic anti-tumor effect of rhein (or diacerein) could be useful in overcoming the resistance to EGFR TKIs and sensitize the EGFR targeted therapy for PC. Rhein or diacerein, when combined with other EGFR targeted agents, may be a novel, clinically accessible STAT3 inhibitor for PC. Thus, our finding could accelerate up the development of clinical therapies by sensitizing human PC cells to EGFR inhibitors through inhibition of STAT3.

## Conclusions

These findings provide for the first time, evidence that rhein exerts antitumor effects by inhibiting the activation of the STAT3 signaling pathway. Our results also suggest that rhein has a promising potential to be used as a novel antitumor agent in cotreatment with EGFR inhibitors. Furthermore, our finding provides new evidence and ideas for targeting STAT3 for the treatment of PC.

## Additional files

Additional file 1: **Figure S1.** Rhein inhibits P-STAT3 and induces apoptosis in pancreatic cancer cell. **(A)** The STAT3 plasmid was transfected into PANC-1 cells and then cells were treated with rhein, P-STAT3 expression was confirmed by Western blotting. **(B)** Cells were treated with rhein at different concentrations as indicated for 36 h, the cell lysates were processed for Western blot analysis for protein expression of BCL-2 and BAX, and the relative intensity was calculated as shown in Fig. 1e. **(C)** Colony forming assay in AsPC-1 cells. Experiments were performed in triplicate and were independently repeated three times. The level of significance is indicated by *P < 0.05, **P < 0.01, and ***P < 0.001 (PDF 159 kb)

Additional file 2: **Figure S2.** Combined rhein and EGFR inhibitors synergistically suppress pancreatic cancer cell proliferation. **(A)** PANC-1 cells were treated with serial dilutions of rhein, the EGFR inhibitor afatinib or the combination of rhein plus afatinib. Cell viability was measured after 3 days of treatment by the MTT assay. CI versus effect curves and isobolograms generated by the calcusyn software. **(B)** The PANC-1 cells were treated with rhein, erlotinib or the combination. CI versus effect curves and isobolograms generated by the calcusyn software. **(C)** The PANC-1 cells were treated with serial dilutions of rhein, gefitinib or the combination. CI versus effect curves and isobolograms generated by the calcusyn software. **(D)** The AsPC-1 cells were treated with serial dilutions of rhein, erlotinib or the combination. CI versus effect curves and isobolograms generated by the calcusyn software (PDF 49 kb)

Additional file 3: **Figure S3.** Combined treatment with rhein and erlotinib inhibit tumor growth in the BxPC-3 xenograft mouse model. **(A)** Antitumor efficacy of rhein and erlotinib in the BxPC-3 xenograft mouse model. BALB/c mice (n = 6) were treated with DMSO (Control), 10 mg/kg erlotinib, 60 mg/kg rhein, or the combination. Tumor volumes were recorded every 2 days. **(B)** Representative images of tumors in each group. **(C)** Comparison of the final tumor weights in each group after the 36-day treatment wtih erlotinib and rhein. Numbers in columns indicate the mean tumor weight in each group. **(D)** Western blot analysis of tumor lysates for phosphorylated EGFR (P-EGFR), phosphorylated STAT3 (P-STAT3), BAX. GAPDH was used as loading control. *p < 0.05, **p < 0.01, ****p < 0.0001 (PDF 189 kb)

## Abbreviations

ADT: AutoDockTools
Af: afatinib
AKT: Protein kinase B
Bax: Bcl-2-associated X protein
Bcl-2: B-cell lymphoma 2
CI: combination index
Cl-PARP: Cleaved poly ADP-ribose polymerase
DAPI: 4, 6-diamidino-2-phenylindole dihydro-chloride
DMSO: dimethyl sulfoxide
EGFR: epidermal growth factor receptor
EMT: epithelial-mesenchymal transition
Er: erlotinib
ERK: extracellular regulated protein kinases
FBS: fetal bovine serum
FDA: Food and Drug Administration
GAPDH: Glyceraldehyde 3-phosphate dehydrogenase
HRP: horseradish peroxidase
IC_50_: the half maximal inhibitory concentrations
IL-6: interleukin- 6
JAK: Janus Kinase
MEK (MAPKK, MAP2K): mitogen-activated protein kinase kinase
MTT: Methylthiazolyldiphenyl-tetrazolium bromide
Napa: Napabucasin
OD: optical density
PBS: phosphate-buffered saline
PC: pancreatic cancer
PDB: Protein Data Bank
PI3K: Phosphoinositide 3-kinase
PVDF: Polyvinylidene Fluoride
Raf (MAPKKK or MAP3K or MEKK): mitogen-activated protein kinase kinase kinase
Rh: Rhein
RPMI: Roswell Park Memorial Institute
SD: Standard deviation
SDS-PAGE: sodium dodecyl sulfate-polyacrylamidegel
STAT3: signal transducer and activator of transcription 3
TKI: tyrosine kinase inhibitors
TKR: tyrosine kinase receptors
