# Supplementary figures and images for: Rhein sensitizes human pancreatic cancer cells to EGFR inhibitors by inhibiting STAT3 pathway
Source: J Exp Clin Cancer Res. 2019 Jan 23;38:31. doi: 10.1186/s13046-018-1015-9 (PMC6343257; doi:10.1186/s13046-018-1015-9)

Figure S1

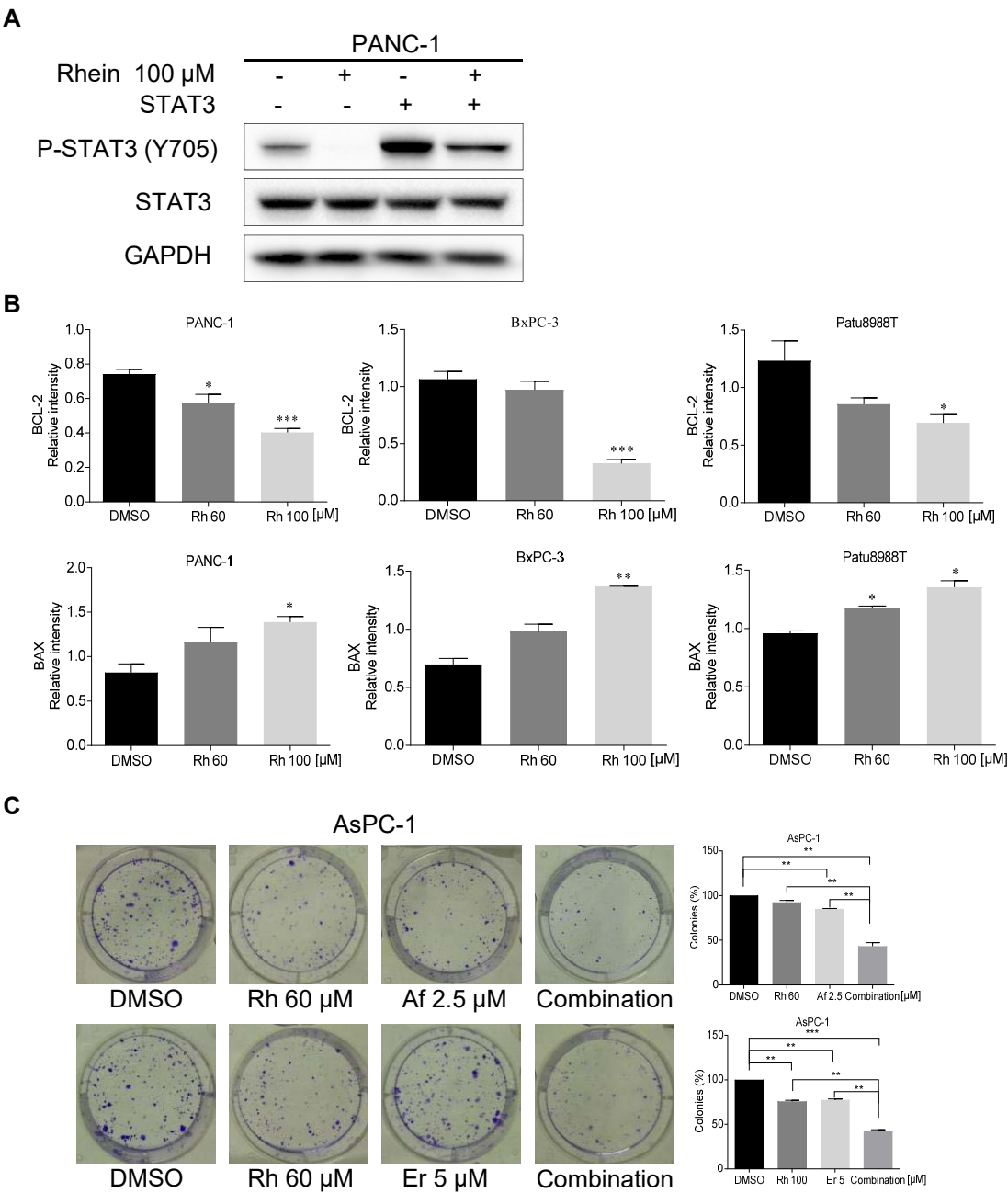

Supplement: Supplementary file 1 — Figure S1. Rhein inhibits P-STAT3 and induces apoptosis in pancreatic cancer cell. (A) The STAT3 plasmid was transfected into PANC-1 cells and then cells were treated with rhein, P-STAT3 expression was confirmed by Western blotting. (B) Cells were treated with rhein at different concentrations as indicated for 36 h, the cell lysates were processed for Western blot analysis for protein expression of BCL-2 and BAX, and the relative intensity was calculated as shown in Fig. 1e. (C) Colony forming assay in AsPC-1 cells. Experiments were performed in triplicate and were independently repeated three times. The level of significance is indicated by *P < 0.05, **P < 0.01, and ***P < 0.001 (PDF 159 kb) [file 13046_2018_1015_MOESM1_ESM.pdf]

Figure S2

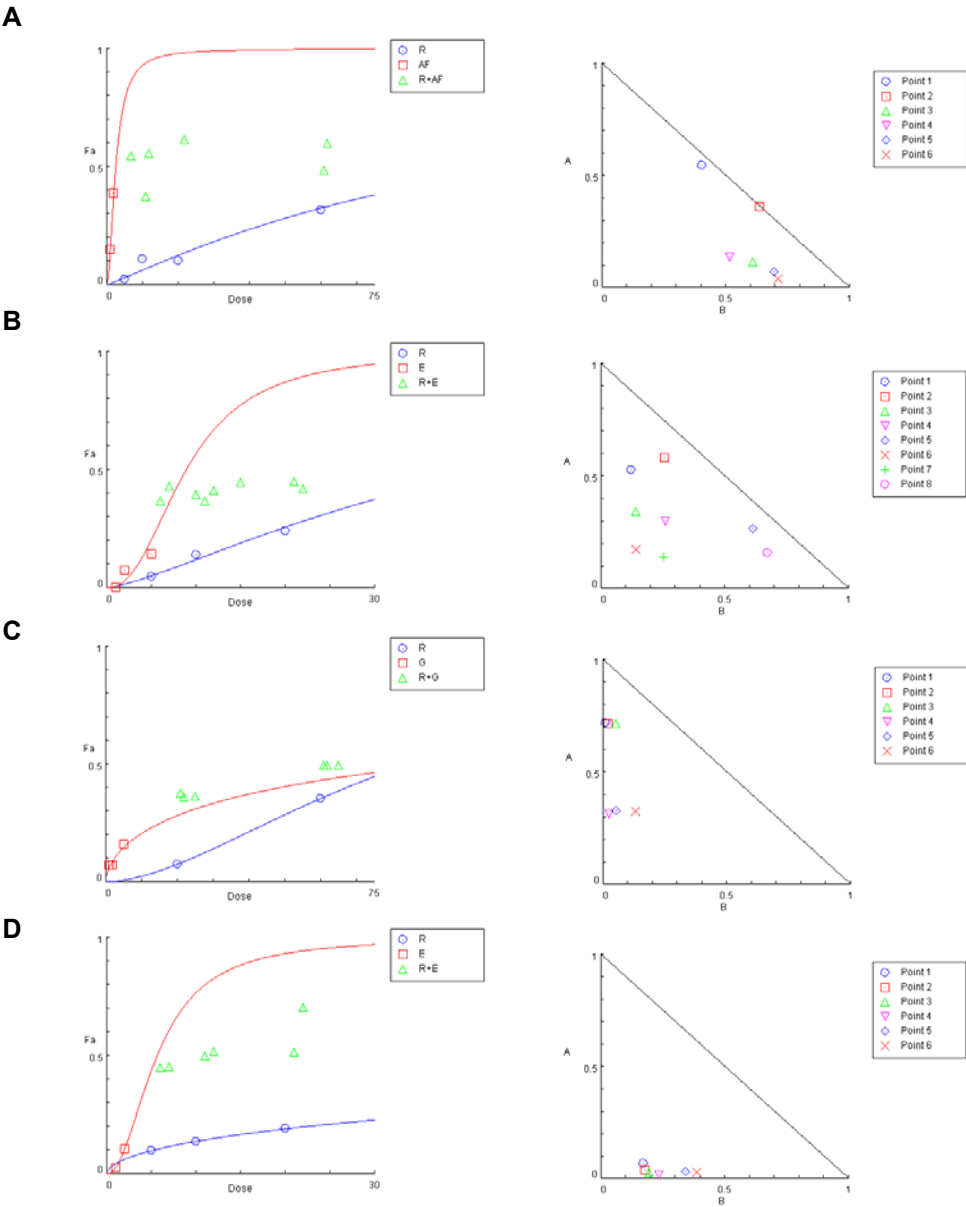

Supplement: Supplementary file 2 — Figure S2. Combined rhein and EGFR inhibitors synergistically suppress pancreatic cancer cell proliferation. (A) PANC-1 cells were treated with serial dilutions of rhein, the EGFR inhibitor afatinib or the combination of rhein plus afatinib. Cell viability was measured after 3 days of treatment by the MTT assay. CI versus effect curves and isobolograms generated by the calcusyn software. (B) The PANC-1 cells were treated with rhein, erlotinib or the combination. CI versus effect curves and isobolograms generated by the calcusyn software. (C) The PANC-1 cells were treated with serial dilutions of rhein, gefitinib or the combination. CI versus effect curves and isobolograms generated by the calcusyn software. (D) The AsPC-1 cells were treated with serial dilutions of rhein, erlotinib or the combination. CI versus effect curves and isobolograms generated by the calcusyn software (PDF 49 kb) [file 13046_2018_1015_MOESM2_ESM.pdf]

Figure S3

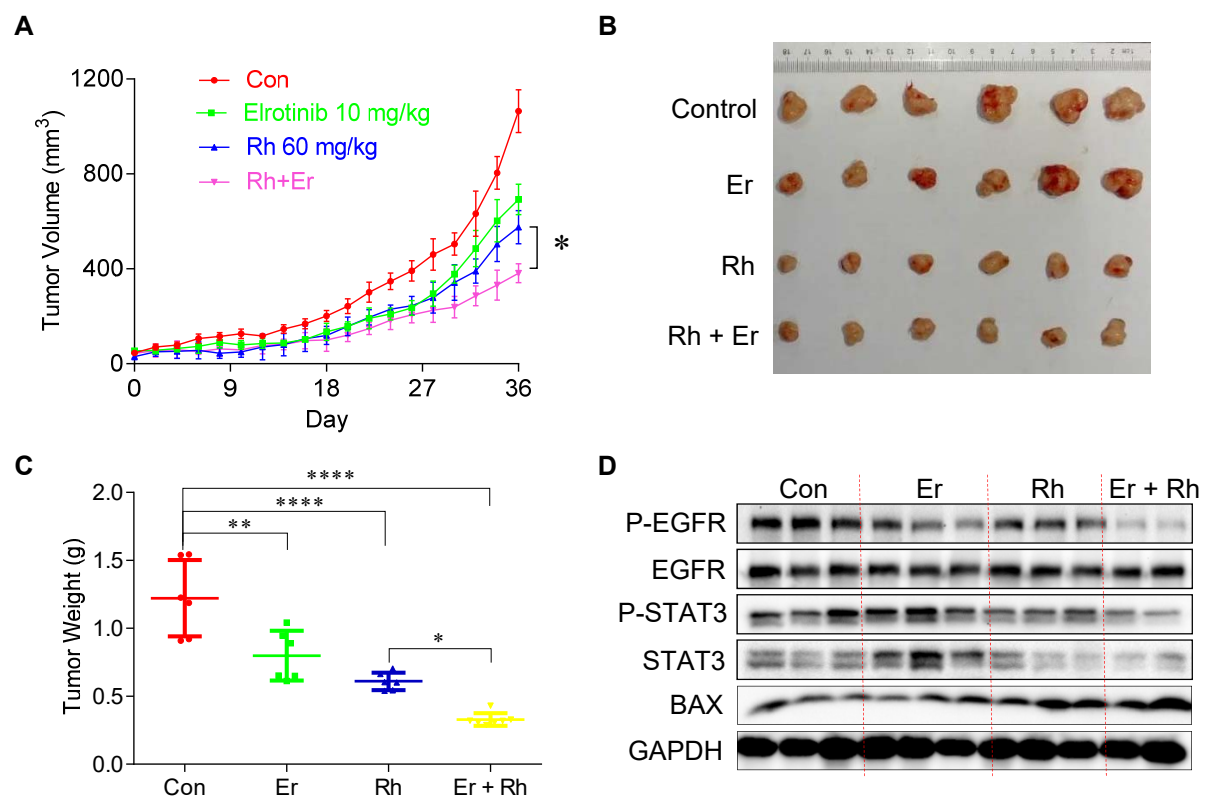

Supplement: Supplementary file 3 — Figure S3. Combined treatment with rhein and erlotinib inhibit tumor growth in the BxPC-3 xenograft mouse model. (A) Antitumor efficacy of rhein and erlotinib in the BxPC-3 xenograft mouse model. BALB/c mice (n = 6) were treated with DMSO (Control), 10 mg/kg erlotinib, 60 mg/kg rhein, or the combination. Tumor volumes were recorded every 2 days. (B) Representative images of tumors in each group. (C) Comparison of the final tumor weights in each group after the 36-day treatment wtih erlotinib and rhein. Numbers in columns indicate the mean tumor weight in each group. (D) Western blot analysis of tumor lysates for phosphorylated EGFR (P-EGFR), phosphorylated STAT3 (P-STAT3), BAX. GAPDH was used as loading control. *p < 0.05, **p < 0.01, ****p < 0.0001 (PDF 189 kb) [file 13046_2018_1015_MOESM3_ESM.pdf]
